# Photothermally Responsive Biomimetic Composite Scaffolds Based on Polydopamine-Functionalized Nanoparticles/Polyurethane for Bone Repair

**DOI:** 10.3390/jfb16080294

**Published:** 2025-08-15

**Authors:** Ruqing Bai, Jiaqi Chen, Ting Zhang, Tao Chen, Xiaoying Liu, Weihu Yang, Tuck-Whye Wong, Jianwei Zhang, Li Wang

**Affiliations:** 1 State Key Laboratory of Mechanical Transmission for Advanced Equipment, Chongqing University, Chongqing 400044, China; ruqing.bai@cqu.edu.cn; 2 School of Big Health and Intelligent Engineering, School of Pharmacy, Chengdu Medical College, Chengdu 610500, Chinaflyrain68@126.com (T.Z.);; 3 Key Laboratory of Biorheological Science and Technology, Ministry of Education, College of Bioengineering, Chongqing University, Chongqing 400044, China; 4 Advanced Membrane Technology Centre, Universiti Teknologi Malaysia, Johor Bahru 81310, Malaysia; 5 Sichuan Provincial Key Laboratory of Philosophy and Social Sciences for Intelligent Medical Care and Elderly Health Management, Chengdu 610500, China; 6 Irradiation Preservation and Effect Key Laboratory of Sichuan Province, Chengdu 610500, China

**Keywords:** 3D scaffolds, shape changing property, bone regeneration, photothermal, biocompatibility

## Abstract

In this study, a shape-changeable 3D scaffold with photothermal effects was developed to address the clinical challenges of complex bone defects. The multifunctional construct was fabricated via in situ polymerization combined with a gas foaming technique, creating hierarchical porous architectures that mimic the native bone extracellular matrix. By incorporating polydopamine (PDA)-modified amorphous calcium phosphate (CA) into poly(propylene glycol) (PPG)- and poly(ԑ-caprolactone) (PCL)-based polyurethane (PU). The obtained scaffolds achieved osteoinductive potential for bone tissue engineering. The surface PDA modification of CA enabled efficient photothermal shape conversion under near-infrared (NIR) irradiation, facilitating non-invasive remote control of localized hyperthermia. The optimized scaffolds exhibited interconnected porosity (approximately 70%) with osteoconductive pore channels (200–500 μm), resulting in good osteoinduction in cell culture, and precise shape-memory recovery at physiological temperatures (~40 °C) under NIR for minimally invasive delivery. The synergistic effect of osteogenesis promotion and photothermal transition demonstrated this programmable scaffold as a promising solution for integrated minimally invasive bone repair and defect reconstruction.

## 1. Introduction

Many patients are afflicted with large segment bone defects, making bone tissue regeneration a vital area of research in both medicine and everyday human life [1]. The conventional treatments for bone defects typically involve the use of bone scaffolds. However, the utilization of autologous and allogeneic bone grafts is constrained by limited availability and the potential risk of immune rejection [2]. Consequently, bone scaffolds have emerged as a promising approach for bone injury treatment due to their abundant sources, renewable nature, and customizable biological properties [3]. An ideal 3D bone engineering scaffold could mimic the microstructure of natural bone, serving as a suitable synthetic extracellular matrix that facilitates cell adhesion and proliferation, ultimately leading to structural reorganization and skeletal reconstruction [4]. Nevertheless, there are still several challenges that need to be addressed, such as the inability to effectively cater to complex bone defect repairs and the issue of mechanical incompatibility with living tissues [5].

For bone repair materials, beyond meeting fundamental structural and functional requirements, clinical applications must prioritize minimizing surgical trauma, reducing wound scarring for postoperative recovery, and restoring aesthetics [6,7]. Shape-memory polymers (SMPs) are intelligent biomaterials that are responsive to external stimuli, achieving predetermined changes in shape [8]. Their shape-memory characteristics exhibit promising potential in minimally invasive surgery. SMPs enable minimally invasive implantation and can adapt to bone defect morphology through pre-programmed shape configurations [9]. Among various stimulation modalities, thermal activation represents the most direct and practically feasible method for in vivo application [10]. The deformation mechanism critically relies on their two-phase architecture comprising crystalline domains and a stationary phase [11]. The initial shape is determined by the stationary phase, while the temporary shape is fixed by the oriented crystalline molecules. However, to achieve precise control of the shape-memory effect (SME) trigger temperature around 37 °C for in vivo applications, functional material selection and structural design are required [12].

Traditional temperature-sensitive SMPs require environmental heating, which limits their applications in biomedical fields, while photothermal composite materials have attracted extensive attention due to their unique properties in converting light energy into heat energy [13]. However, some inorganic metal complexes have poor biocompatibility and may cause toxic side effects in the body [14]. In addition, some materials have low photothermal conversion efficiency and poor stability under repeated thermal cycling [15]. Nevertheless, polydopamine (PDA) has emerged as a promising material to address these limitations [16], owing to its excellent adhesive properties and ability to firmly bind to other materials [17]. It also has photothermal effects, which can convert light energy into heat energy, producing a local temperature increase [18]. This property can promote blood circulation in the local area, accelerate the delivery of nutrients and the removal of metabolic waste, and thus promote bone tissue repair and regeneration [19,20]. The PDA-modified materials can enhance photothermal performance, making them more suitable for minimally invasive surgery and bone defect repair.

**Figure 1:**
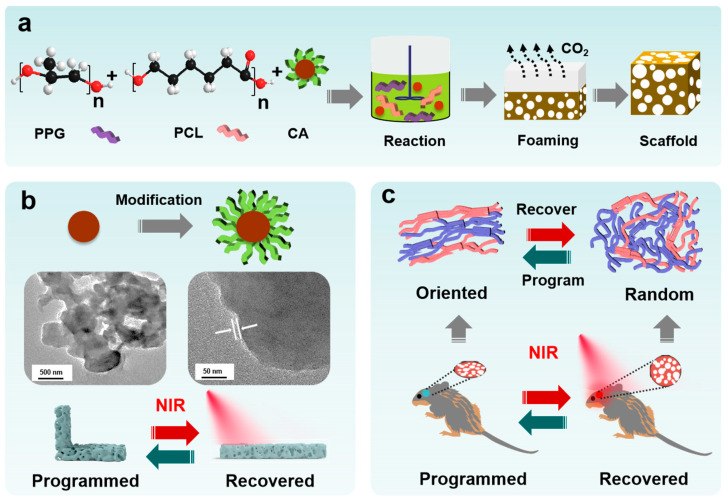
(**a**) Schematic representation of preparation process for PUCA smart scaffold; (**b**) the surface modification of CA by PDA and the TEM images; (**c**) the molecular structure of shape programming/recovering process and the minimally invasive implantation.

In this study, smart shape-memory polymer composite scaffolds are prepared by the gas foaming method, and the polymer matrix is composed of elastic PPG and biodegradable PCL as the soft segment, with crosslinking by aliphatic hexamethylene diisocyanate (HDI) to develop interconnected pores during the post-curing process (Figure 1a). The PDA-modified CA can contribute to the photothermal performance of the composite material, resulting in a programmable pore structure, making it more suitable for minimally invasive surgery and bone defect repair (Figure 1b,c). The composite scaffolds not only present excellent photothermal conversion efficiency but also exhibit good biocompatibility and osteoinductivity. Biomineralization and osteogenesis differentiation are evaluated by in vitro assessment in mesenchymal stem cells (MSCs). Typically, their in vivo biocompatibility is evaluated by animal experiments.

## 2. Experimental Section

### 2.1. Materials

Raw materials were purchased from Jining Hua Kai Resin Co., Ltd., Jining, China, including poly(propylene glycol) (PPG, M_n_~3000 g/mol) and poly(ԑ-caprolactone) (PCL, M_n_~3000 g/mol), both with PDI~2. Tin 2-ethylhexanoate (SnOct2, >95.0%) and hexamethylene diisocyanate (HDI, C8H12N2O2, >99.0%) of AR grade were purchased from Aladdin Chemical Reagent Factory (Shanghai, China).

### 2.2. Synthesis of PDA-Modified Amorphous Calcium Phosphate (CA)

Amorphous calcium phosphate (CA) nanoparticles were synthesized via a precipitation approach. At room temperature, 16.647 g of calcium chloride (CaCl_2_) was dissolved in water to obtain a 300 mL aqueous solution. Next, 14.705 g of trisodium citrate dihydrate and 6.603 g of diammonium hydrogen phosphate (DAP) were combined to form a 200 mL solution, which was then transferred to a dropping funnel. Successively, 300 mL of solvent (water/ethanol volume ratio = 2:1) was prepared and placed in the funnel. During the titration process, mixed solutions were gradually added to the calcium chloride solution under continuous pH 10 using aqueous ammonia. The obtained precipitate was aged with daily renewal of the supernatant for one week. The sediment underwent three cycles of washing with deionized water, followed by absolute ethanol. For surface modification, a Tris buffer solution (pH adjusted to 10 with 0.1 M HCl) was prepared, into which dopamine hydrochloride powder was introduced to achieve a final concentration of 2 mg/mL. Varying amounts of nanoparticles were subsequently added to the solution. The mixture was stirred in darkness at ambient temperature for 12 h, followed by a 3-day static aging period with daily supernatant replacement. Finally, the product was collected via centrifugation, lyophilized, and ground into powder for characterization. Finally, the nanoparticle morphology was examined using a transmission electron microscope (TEM, TECNAI G2 F20S-TWIN, Hillsboro, OR, USA) operated at 30 kV, after dispersing the samples in absolute ethanol.

### 2.3. Preparation of PUCA Scaffolds

Firstly, by the co-precipitation method, amorphous calcium phosphate nanoparticles (CA) were synthesized [21]. Subsequently, dopamine hydrochloride powder was added to the Tris buffer solution. Finally, the mixture was centrifuged, the supernatant was removed, and the precipitate was separated and dried at 60 °C. Afterwards, 20 g PPG and PCL were added together with 50 mL of dichloromethane, followed by adding various weight contents of CA. After stirring at a temperature of 80 °C under a nitrogen atmosphere, HDI and SnOct_2_ were added to react for 3 h. The reaction mixture was poured into a polytetrafluoroethylene mold, curing overnight at 120 °C after adding a foaming agent for 12 h in an oven. The prepared composite scaffold materials were named PUCAx, where x stands for the weight ratio of CA.

### 2.4. Characterization of Physical and Chemical Properties of PUCA Scaffolds

The infrared spectrum of PUCA was characterized by a Fourier transform infrared spectrophotometer (FTIR, TENSOR-27, Bruker, Germany). The crystalline structure of PUCA was analyzed by X-ray diffraction (XRD, DX-2700, Fangyuan Instrument Co., Ltd., Wenzhou, China). The micro-scale structure of the PUCA was analyzed by scanning electron microscopy (FE-SEM, INSPECTF50, FEI, Eindhoven, The Netherlands). The distribution of the nanoparticles in the smart scaffold was characterized by energy dispersive spectroscopy (EDS). The porosity was evaluated by the Archimedes principle. First, the dry sample was weighed and recorded as M_1_. Next, the sample was put in water for 24 h, and the swollen sample was weighed and recorded as M_2_. In the end, the saturated sample was weighed in water and recorded as M_3_.

(1) $$\mathrm{Porosity}\;(\%)=\frac{M_{3}-M_{1}}{M_{3}-M_{2}}\times 100\%$$

(2) $$\mathrm{Density}\;(g/{\mathrm{cm}}^{3})=\frac{M_{1}}{M_{3}-M_{2}}$$

Young’s modulus was characterized by a universal testing machine (MIT-30KN, Changzhou SFMIT apparatus Co., Ltd., Changzhou, China), with a size of 10 × 10 × 10 mm (ASTM standard D695–96). Additionally, wettability is measured as the water contact angle 5 s after the droplet landing, using standardized methods (ISO 19403-2) and ensuring environmental stability (25 °C, 40–60% RH). Measurements were recorded within 3 s post-stabilization to minimize evaporation errors and were repeated 3 times. Moreover, the swelling degree (S) and gel content (G) were characterized by swelling in ethyl acetate for one day. The weights of the initial (W_0_), swollen (W_1_), and dried samples (W_2_) were weighed and recorded, and they are calculated as follows:

(3) $$S\;(\%)\;=\;\frac{W_{1}-W_{0}}{W_{0}}\times 100\%$$

(4) $$G\;(\%)=\frac{W_{2}}{W_{0}}\times 100\%$$

### 2.5. Thermal Behavior Analysis of PUCA Scaffolds

The differential scanning calorimeter (DSC, NETZSCH DSC-204F1) of PUCA samples was measured starting at −50 °C and ending at 100 °C, with a temperature change rate of 10 °C/min, and thermogravimetric analysis (TGA, STA-409-PC, NETZSCH, Selb, Germany) was performed from 25 °C to 800 °C at a temperature change rate of 10 °C/min.

### 2.6. Shape-Memory Properties Analysis of PUCA Scaffolds

PUCA scaffolds’ shape-memory performance was evaluated with bending experiments [21]. Specifically, L-shaped samples (*n* = 3) were heated up to 50 °C for 10 min and then bent; next, they were cooled down to 0 °C to keep the original shape. Between the original sample and the vertical one, the angle change was θ_t_, while the programmed and recovered angles were recorded as θ_p_ and 90°, respectively. The shape recovery rate (R_r_) and shape-memory fixity (R_f_) were calculated as follows:

(5) $$R_{f}\;(\%)\;=\;\frac{\theta_{p}}{90{}^{\circ}}\times 100\%$$

(6) $$R_{r}\;(\%)=\frac{\theta_{t}}{90{}^{\circ}}\times 100\%$$

To simulate physiological shape-memory stress evolution during the process, a user-defined subroutine was processed in ANSYS 2022 R1, incorporating a visco-hyperelastic constitutive model. Viscoelastic properties were modeled by the Maxwell framework, followed by the Neo-Hookean equation for hyperelasticity. The simulation stretched the model to 130% of its original length, ranging from 25 to 60 °C. This experimental/computational simulation characterized the thermal/mechanical shape-memory properties, enabling predictive analysis of biomedical device performance under physiological conditions.

### 2.7. In Vitro Cytocompatibility Evaluation of PUCA Scaffolds

The in vitro biocompatibility of scaffolds and cell proliferation were evaluated using MSCs (1 × 10^4^ cells/mL, *n* = 3) co-cultured with extracts (0.2 g/mL) prepared from sterilized scaffolds soaked in α-MEM, following ISO 10993-5:2009 guidelines. The cell lines of MSCs were from direct harvesting of femurs and tibias of SD rats. MSCs were isolated from 3-week-old SD rat femurs. Proliferation was compared to a control group (F-12 medium) via a CCK-8 assay at 1, 3, and 5 days. Optical density (OD) was observed by a microplate reader (PerkinElmer, Shelton, CT, USA) at 450 nm. Additionally, osteoblastic differentiation was evaluated for PUCA scaffolds. Cell viability assessment was conducted after 4 and 7 days of culture. Adherent cells were gently rinsed with sterile phosphate-buffered saline (PBS) and incubated with MTT reagent (3-(4,5-dimethylthiazol-2-yl)-2,5-diphenyltetrazolium bromide) under standard culture conditions for 2 h. The formation of formazan crystals, indicative of mitochondrial activity, was quantified using absorbance values at 490 nm with a Bio-Rad 680 microplate reader. For cytoskeletal analysis, mesenchymal stem cells (MSCs) were seeded at 1 × 10^4^ cells/mL in 24-well plates and cultured with test extracts for 7 days. Cells were fixed with 4% paraformaldehyde solution, followed by nuclear staining with Hoechst 33258 dye for 10 min. Confocal laser scanning microscopy (Leica TCP SP5, Mannheim, Germany) was employed to visualize cellular morphology. Osteogenic differentiation was evaluated through alkaline phosphatase (ALP) activity, a key biomarker for osteoblastogenesis. MSCs (1 × 10^4^ cells/mL, *n* = 3) were cultured for 4 and 7 days, and then processed for enzymatic quantification using a commercial ALP kit (Bekotime, Shanghai, China). Following cell lysis with 1% Triton X-100, the lysates were incubated with ALP substrate solution for 2 h. Absorbance was recorded at 490 nm using the same microplate reader. Concurrent histological analysis was performed using a BCIP/NBT ALP color development kit, with visualization via MVX10 MacroView, Optoelectronics Technology Co., Ltd., Tokyo, Japan. Collagen type I deposition was assessed after 7 days of MSC culture with scaffold extracts. Cultures were stained with Sirius Red solution for 1 h, followed by qualitative imaging using the Olympus MVX10 system. For quantitative analysis, stained samples were treated with sodium hydroxide solution, and collagen content was determined by measuring absorbance at 540 nm with the Bio-Rad 680 platform.

### 2.8. In Vivo Assessment of PUCA Scaffolds

In vivo assessment was evaluated by observing the osteogenic capacity of scaffolds using a rat cranial defect model for observing bone regeneration. Animal experiments were approved by the Chongqing University Institutional Animal Care and Use Committee (CQU-IACUC-RE-202205-001, May 2025). Specifically, 4-week-old male SD rats (Daping Hospital, Chongqing, China) were anesthetized, and their skulls were exposed via shaving and incision. Using a trephine bur under sterile saline irrigation, two 5 mm round wounds were created in every skull. One defect remained untreated (control), while the other was implanted with a cylindrical sample (5 mm × 1 mm). Rats were sacrificed at 2, 6, and 12 weeks using chloral hydrate. Histological analyses were performed and included H&E, Masson’s trichrome, ALP, and IHC staining to assess cell infiltration, collagen deposition, and osteogenic differentiation. Additionally, bone regeneration at various times was evaluated by micro-computed tomography (SkyScan 1276, Bruker, Singapore). Bone regeneration parameters were quantified by three-dimensional reconstructions (N-Recon software, version 1.7.0.5) and analyses (CT-AN software, version 1.15), including bone volume/tissue volume (BV/TV) and trabecular number (Tb.N).

### 2.9. Statistical Analysis

Data are presented as mean ± standard deviation (SD). Statistical evaluations were conducted using independent samples t-tests and one-way analysis of variance (ANOVA) via SPSS Statistics version 24.0. Statistical significance was defined at * *p* < 0.05.

## 3. Results and Discussion

### 3.1. Synthesis and Structure Characterization of PUCA Scaffolds

**Figure 2:**
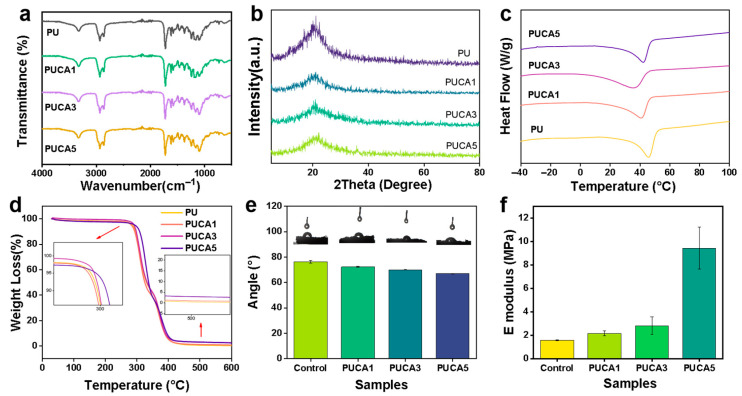
(**a**) FT-IR spectra of PUCA scaffolds; (**b**) XRD pattern of PUCA scaffolds; (**c**) DSC thermograms of 2nd heating cycle for PUCA scaffolds; (**d**) TG curve of PUCA scaffolds with various CA content; (**e**) water contact angle of PUCA scaffolds (*n* = 3); (**f**) Young’s modulus of PUCA scaffolds (*n* = 3).

The successful synthesis, conformation, and chemical structures of the PUCA scaffolds were verified by FT-IR, as shown in Figure 2a. An absorption peak at 3340 cm^−1^ was detected, corresponding to the stretching vibration of the N-H bond. Moreover, the peaks at 2853 cm^−1^ and 2937 cm^−1^ were attributed to the asymmetric and symmetric stretching vibrations of the -CH_2_ group. No characteristic peaks of the -NCO group were found in the range of 2260–2280 cm^−1^, proving the complete reaction of hexamethylene diisocyanate (HDI). In addition, the absorption peak at 1700 cm^−1^ originated from the C=O stretching vibration of urethane, while the C-N peak at 1462 cm^−1^ and the C-O-C peak at 1100 cm^−1^ confirmed the formation of polyurethane [22]. The characteristic PO_4_^3−^ absorption peak near 575 cm^−1^ gradually intensified with increasing CA content, demonstrating successful incorporation of the nanoparticles into the composite system. The XRD patterns in Figure 2b show a broad diffraction peak near 21°, typical of polymers with coexisting crystalline and amorphous regions [23,24]. As CA content increased from 1 wt% to 5 wt%, the typical crystalline peaks gradually emerged, while the diffraction intensity of the polymer matrix decreased overall, indicating effective integration of the functional nanoparticles.

The melting temperature (T_m_) of polyurethanes serves as a trigger switch [25], critical for the smart scaffolds’ shape recovery. The DSC results (Figure 2c) revealed that Tm decreased from 45.83 °C to 37.17 °C with CA addition, due to disturbing the crystalline order in PU. PUCA5 exhibited a slightly higher Tm than other samples, likely due to the nucleation effects of trace nanoparticles enhancing PU crystallization. Thermogravimetric analysis (Figure 2d) showed two thermal degradation stages, i.e., first, the decomposition of PCL and urethane bonds at 270–350 °C, followed by polypropylene glycol (PPG) degradation from 350 to 400 °C [26]. The Tm of PUCA scaffolds confirmed good thermal stability, fulfilling the requirements for shape-memory polymeric scaffolds [27]. Figure 2e shows the average contact angles of porous composites with different ratios. Because of the hydrophilic CA, the contact angles of the scaffolds decreased as the CA content increased. In contrast, with the increase in CA content, the E-modulus increased from 1.7 MPa to 9.5 MPa, as shown in Figure 2f.

**Figure 3:**
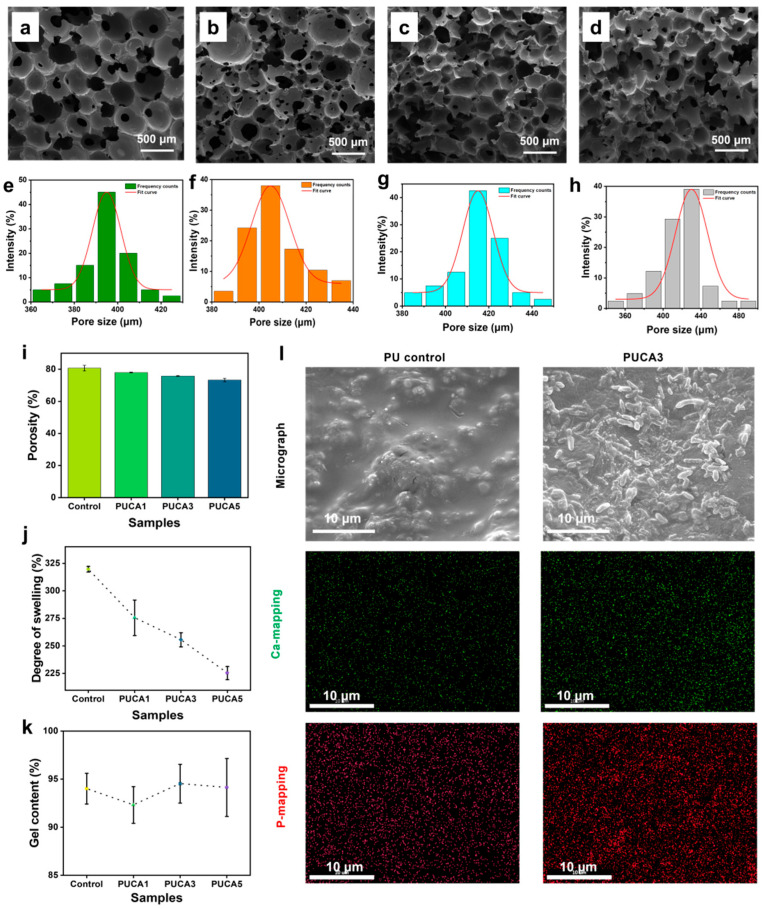
(**a**–**d**) SEM images and (**e**–**h**) pore size distribution of PUCA scaffolds with (**i**) porosity, (**j**) gel content, and (**k**) degree of swelling of the PUCA scaffolds. (**l**) SEM images of different samples after 14 days of SBF immersion, and EDS images and element distribution of PUCA scaffolds.

The pore structure critically influences material performance and application efficacy, serving as a key indicator for evaluating bone tissue engineering scaffolds. An appropriate pore structure provides three-dimensional support and guidance, facilitating directional cell growth and tissue regeneration [28,29]. SEM images of PUCA scaffolds are shown in Figure 3a–d. All scaffolds exhibited well-defined pore architectures. More specifically, Figure 3e–h illustrate the pore size distribution of PUCA; with increasing CA filler content, the pore diameters slightly increased by 395 ± 10 µm, 405 ± 20 µm, 425 ± 10 µm, and 430 ± 10 µm, respectively. A cell diameter of around 100–500 µm is highly favorable for cell adhesion and proliferation. The absence of visible large particles on the material surface indicates that nanoparticle incorporation did not significantly interrupt the microporous structure of the composite scaffolds, confirming uniform distribution and excellent interfacial compatibility between CA and the PU matrix.

The porosity of the PUCA scaffolds was measured and presented (Figure 3i). As filler content increased from 1 wt% to 5 wt%, porosity decreased from 80.71% ± 1.17% to 73.28% ± 1.09%, with the interconnectivity dropping. This was attributed to increased polyurethane viscosity upon filler addition, hindering CO_2_ escape during foaming, resulting in thicker pore walls and decreased connectivity [30]. Nevertheless, all samples maintained excellent porosity (>70%) and interconnectivity (>80%), satisfying requirements for bone repair applications [31]. Using ethyl acetate as the medium, the swelling ratio and gel content of PUCA scaffolds were furtherly measured, as shown in Figure 3j,k. All samples exhibited high gel content (>90%), indicating stable crosslinked networks. Notably, the swelling ratio decreased from 319.64% ± 2.59% to 225.36% ± 5.95% with increasing CA content, due to physical crosslinking induced by fillers restricting molecular chain mobility.

To evaluate the mineralization activity of PUCA scaffolds, PUCA3 samples were immersed in simulated body fluid (SBF). SEM images and EDS elemental mapping of calcium (Ca) and phosphorus (P) are shown in Figure 3l. The SEM images revealed significant HA deposition on the scaffold surface, indicating that the CA fillers effectively promoted HA formation, primarily via amorphous calcium phosphate (ACP) transformation into HA in SBF. Additionally, the EDS results showed uniform distribution of Ca and P elements, with increased concentration and homogeneity upon filler incorporation. PUCA3 demonstrated superior mineralization activity, creating optimal conditions for new bone regeneration. These results proved that the composite scaffolds could enhance the bioactivity and promote osteogenesis.

### 3.2. Shape-Memory and Photothermal Effect of PUCA Scaffolds

**Figure 4:**
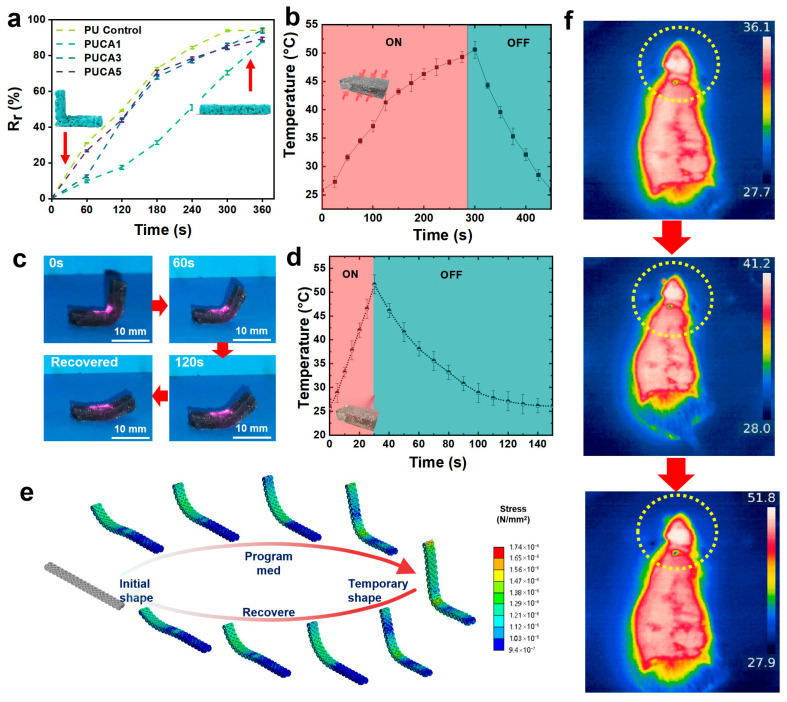
(**a**) Shape recovery rate versus time for PUCA scaffolds during compression cycles (*n* = 3); (**b**) temperature changing curve of shape-memory transition switch of scaffolds triggered heating by an oven; (**c**) shape-memory transition switch heating by NIR; (**d**) temperature changing curve in NIR; (**e**) shape-memory process realization of scaffolds using finite element simulations; (**f**) infrared images of implanted rats heated by NIR with different temperatures, where heating areas marked with dotted line circles.

To evaluate the shape-memory properties of PUCA, bending tests were conducted on the samples. The shape-memory process was illustrated. As shown in Figure 4a, the PUCA sample, originally in a horizontal state and subsequently fixed into a vertical configuration, was placed in a 50 °C oven for direct heating. The sample gradually recovered its original shape, showing excellent shape fixation and recovery efficiency. By statistically analyzing the angular changes during shape recovery, the shape fixity ratio and recovery ratio of the PUCA bone scaffolds were calculated, with the results presented in Figure 4a. The time for the completion of shape recovery of PUCA samples was prolonged compared to pure PU samples due to the incorporation of CA fillers. When the filler content increased from 0 wt% to 5 wt%, the shape recovery ratio decreased from 93.9% to 89.2%. This phenomenon was attributed to the disruption of material crystallinity and hindrance of chain mobility from nanoparticles, preventing the molecular chains from freely transitioning from oriented to random states, thereby slowing down the shape recovery rate [32]. To further investigate the photothermal effects of PUCA bone scaffolds containing varying mass fractions of photo-responsive CA nanoparticles, the PUCA3 sample was irradiated with NIR light (0.2 W/cm^2^) and its temperature–time profile was measured using an infrared thermal imaging camera, as shown in Figure 4c. Compared to samples heated directly in an oven (Figure 4b), NIR-irradiated samples demonstrated significantly accelerated heating rates, reaching 40 °C within 25 s to trigger shape-memory effects and initiate recovery, and attaining their critical temperature within 30 s (Figure 4d). This rapid heating was attributed to the excellent photothermal conversion capability of PDA. The uniform dispersion of CA@PDA fillers in the PUCA matrix enabled efficient heat transfer throughout the material.

To further investigate the photothermal response of PUCA bone scaffolds to remote NIR irradiation in vivo, animal simulation experiments were conducted. The visualized shape recovery process under NIR irradiation is presented in Figure 4f. PUCA1, PUCA3, and PUCA5 samples were implanted into mouse skulls, and the implantation sites were irradiated with NIR (0.2 W/cm^2^) while monitoring temperature changes via infrared thermal imaging (Figure 4f). All three PUCA samples with varying filler contents exhibited exceptional photothermal effects under NIR irradiation, rapidly reaching critical temperatures (above 50 °C) within 30 s. This not only triggered the shape-memory effect of PUCA bone scaffolds for complete shape recovery and bone defect matching but also satisfied the temperature requirements. The PUCA bone scaffold material combines excellent physicochemical properties with multi-responsive (light and heat) shape-memory effects and photothermal capabilities, holding significant implications for tissue engineering. Finally, infrared images from implanted rats heated by NIR at different temperatures were taken, and the findings provide favorable data for the remote NIR-responsive shape-memory recovery of PUCA bone scaffolds post-implantation, demonstrating significant application potential.

### 3.3. In Vitro and In Vivo Biological Compatibility Assessment of PUCA Scaffolds

**Figure 5:**
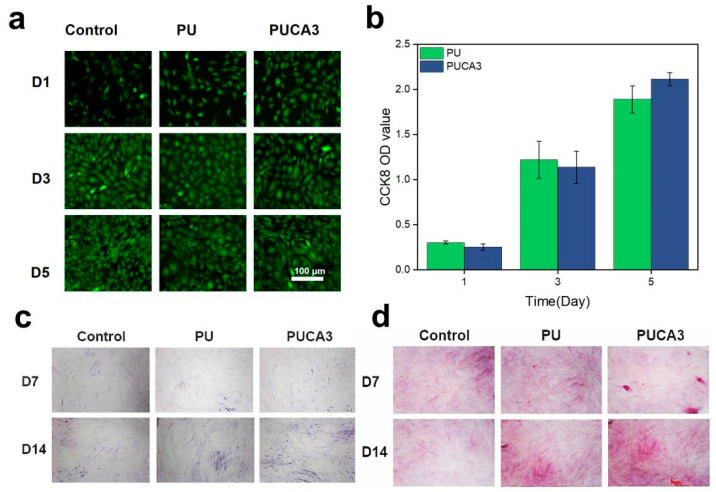
(**a**) Fluorescence photos of MSCs cultured in PUCA extracted solution for 1, 3, and 5 days; (**b**) cell viability of MSCs measured at the surface of PU and PUCA3 after 1, 3 and 5 days; (**c**) ALP activity and representative pictures of ALP staining of MSCs after 7 and 14 days of surface culture the scaffolds; (**d**) representative fluorescence quantification pictures of Col I in MSCs cultured in PU and PUCA3 for 7 and 14 days.

In this study, CA particles can provide exogenous calcium ions to regulate the proliferation and differentiation of various cells during the bone repair phase and modulate osteogenesis by promoting neovascularization at bone defects and stimulating the release of growth factors. Additionally, the bioactivity of PDA enhances cell adhesion and proliferation on its surface. To further investigate the biocompatibility of the PUCA bone scaffold, the CCK-8 kit was used to assess cell proliferation and cytotoxicity on the material. Live/dead fluorescence staining was performed on MSCs cultured on the material, with the results shown in Figure 5a, where green and red fluorescent dots represent live and dead cells, respectively. MSCs exhibited good growth on the material after 3 days, with a directional spread and nearly complete coverage of the substrate surface. On day 5, PUCA3 cells showed pseudopod extension, and quantitative analysis (Figure 5b) indicated that their cell viability was stronger than that of PU material, likely benefiting from the bioactivity of CA@PDA.

Alkaline phosphatase (ALP) is involved in the process of bone mineralization in skeletal tissue, converting inorganic phosphates into phosphates, thereby providing the necessary phosphorus source for bone mineralization. ALP is considered a marker of osteoblast maturation and function. Increased expression of ALP indicates enhanced osteoblast differentiation and activity, thereby promoting bone tissue growth and repair. ALP staining images on days 4 and 7 are shown in Figure 5c, revealing that the ALP activity of the PUCA3 sample was significantly higher than that of the control group, with superior ALP expression compared to the PU sample. This is attributed to the promotive effect and bioactivity of CA on bone regeneration, demonstrating that the PUCA3 bone scaffold provides a favorable environment for bone repair in the early stages of osteogenic differentiation. Type I collagen (Col I) is an extracellular matrix protein primarily found in tissues such as bone, tendons, ligaments, and blood vessels. Its main function is to provide structural support and strength to tissues, enabling them to maintain stable morphology and function. Furthermore, Col I is involved in physiological processes such as wound healing, bone growth, and repair. To further investigate the genetic expression of osteoblasts, MSCs were cultured on PU and PUCA3 samples for 7 and 14 days, respectively, and Col I expression was analyzed. Representative fluorescence staining images of the samples (Figure 5d) show that both PUCA3 and PU samples formed certain mineralized calcium nodules, indicating that the addition of PDA-modified nanofillers does not reduce the osteoinductivity of the scaffolds.

**Figure 6:**
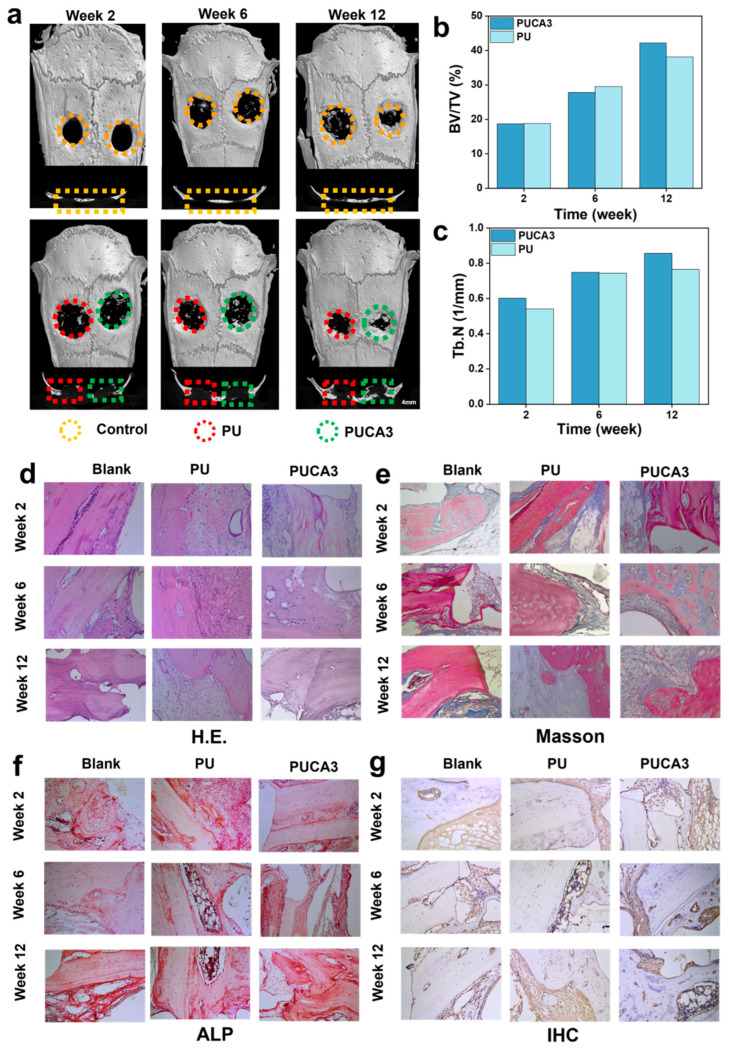
(**a**) Micro-CT reconstruction images of defect area with surrounding tissue; (**b**) morphometric analysis of bone volume/total volume (BV/TV); (**c**) trabecular number (Tb.N) for porous scaffolds; histochemical staining of scaffolds from week 2 to week 12 by (**d**) H.E., (**e**) Masson, (**f**) ALP, and (**g**) IHC.

To further investigate the in vivo osteogenic repair effect of the PUCA3 scaffold, the samples were implanted into a critical-sized mouse calvarial defect model, and three-dimensional imaging reconstruction using Micro-CT was performed on the implantation sites at 2, 6, and 12 weeks. The PU and PUCA3 bone scaffolds were implanted into the left and right bone defect regions of the mouse calvaria, respectively, to study their bone repair outcomes (Figure 6a). Quantitative evaluations of bone volume fraction (BV/TV) and trabecular number (Tb.N) were conducted, as shown in Figure 6b,c. As demonstrated, the PU group exhibited only minimal bone regeneration at 2, 6, and 12 weeks, whereas the implantation site of the PUCA3 group showed more new bone formation with higher bone density than the PU group. This indicates that PUCA3 exhibits superior bone repair effects and can promote the formation of more new bone. The quantitative analysis results are consistent with the imaging trends, showing that PUCA3 promotes the formation of more trabeculae. At 2 and 6 weeks post-implantation, the bone volume fraction was slightly lower than that of the PU group, but in the later stage of implantation, at 12 weeks, the PUCA3 sample demonstrated good osteogenic properties, with both BV/TV and Tb.N higher than those of the PU group.

Figure 6d–g show histological staining sections at different time points (2 weeks, 6 weeks, and 12 weeks post-implantation). H&E staining (Figure 6g) shows more cellular infiltration, while Masson staining (Figure 6e) shows more collagen fiber deposition in the PUCA3 group at 12 weeks, indicating more mature and stable new bone tissue. By focusing on specific areas, the ALP (Figure 6f) and IHC staining (Figure 6g) show detailed structures of trabeculae and bone cells, and the PUCA3 group shows richer trabeculae and more mature bone tissue. These results indicate that the PUCA3 bone scaffold outperforms the PU scaffold in vivo for bone repair. PUCA3 promotes more new bone formation, increases bone density, and enhances trabecular number, demonstrating better osteogenic properties. These findings support the application of PUCA3 bone scaffolds in bone defect repair.

## 4. Conclusions

In this study, a multifunctional 3D scaffold was developed with photothermal responsiveness and integrated therapeutic functions for bone tissue damage. The surface of CA with PDA modification enabled efficient photothermally conversion under near-infrared (NIR) irradiation, facilitating non-invasive remote control of localized shape-changing capability. The optimized PUCA scaffold demonstrated interconnected porosity with osteoconductive pore channels, which supported good osteoinduction in cell culture. Additionally, the scaffold showed precise shape-memory recovery at physiological temperatures (~40 °C) under NIR, facilitating minimally invasive delivery. The synergistic effect of osteogenesis promotion and photothermal therapy demonstrated this programmable scaffold as a transformative solution for bone defect reconstruction. The limitation of the scaffolds might be the inefficient penetration ability, which might be improved by changing to magnetically induced power. The excellent biocompatibility, high photothermal conversion efficiency, and shape-memory properties of the PUCA scaffold make it a promising candidate for bone tissue engineering applications.

## Data Availability

All data generated or analyzed during this study are included in this article.
